# The Relationship Between Body Composition Parameters and the Intake of Selected Nutrients, and Serum Anti-Müllerian Hormone (AMH) Levels in the Context of Ovulatory Infertility

**DOI:** 10.3390/nu16234149

**Published:** 2024-11-29

**Authors:** Magdalena Skowrońska, Michał Pawłowski, Angelika Buczyńska, Aleksandra Wiatr, Aleksandra Dyszkiewicz, Aleksandra Wenta, Kamila Gryko, Monika Zbucka-Krętowska, Robert Milewski

**Affiliations:** 1 Doctoral School, Medical University of Bialystok, 15-276 Bialystok, Poland; magdalena.skowronska@sd.umb.edu.pl; 2 Department of Biostatistics and Medical Informatics, Medical University of Bialystok, 15-295 Bialystok, Poland; michal.pawlowski@umb.edu.pl; 3 Clinical Research Center, Medical University of Bialystok, 15-276 Bialystok, Poland; angelika.buczynska@umb.edu.pl (A.B.); aleksandra.wiatr19@gmail.com (A.W.); 4 Department of Endocrinology, Diabetology and Internal Medicine, Medical University of Bialystok, 15-276 Bialystok, Poland; aleksandra.dyszkiewicz@umb.edu.pl; 5 Department of Gynecological Endocrinology and Adolescent Gynecology, Medical University of Bialystok, 15-276 Bialystok, Poland; alewenta@gmail.com (A.W.); kamilagryko@gmail.com (K.G.); monika.zbucka-kretowska@umb.edu.pl (M.Z.-K.)

**Keywords:** AMH, ovulatory infertility, body composition, nutrients, dietary advice

## Abstract

**Background/Objective:** The aim of this study was to outline the relationships between selected parameters connected with lifestyle and serum anti-Müllerian hormone (AMH) levels, i.e., a marker of ovarian reserve. By examining AMH levels in connection with nutrient intake and body composition parameters, this study aimed to provide a preliminary background for further studies focused on establishing dietary and lifestyle recommendations that could lead to improvements in fertility outcomes. **Methods:** The research involved 28 women, aged 26 to 42—both with and without ovulatory infertility—who were patients of the Reproductive Health Clinic at the Medical University of Białystok. The participants underwent a number of tests consisting of hormonal profiling, including AMH measurements, body composition analyses, and dietary assessments based on a 3-day food diary. **Results:** The findings of the study indicate that certain lifestyle factors are associated with changes in AMH levels. Most importantly, the multivariate linear regression model designed in the study shows that age, waist-to-hip ratio (WHR), as well as the intake of sucrose, iodine, and erucic acid explain variations in serum AMH levels. These results support the hypothesis that modifiable lifestyle factors can influence AMH levels, and thus ovarian reserve. **Conclusions:** The study underscores the potential for targeted lifestyle interventions to support fertility and calls for further research in the form of prospective studies performed in larger groups of patients to substantiate these associations and inform fertility care strategies. Based on the preliminary results of this study, certain dietary ideas that could positively influence fertility have been proposed, focused on the normalization of body weight and the reduction in excess fat tissue.

## 1. Introduction

Infertility, as defined by the World Health Organization (WHO), is a disease affecting the male or female reproductive system, characterized by the inability to achieve pregnancy after 12 months or more of regular, unprotected sexual intercourse [1]. Globally, infertility affects an estimated 50 to 70 million couples [2], with around half of these cases attributed to female reproductive issues. While clinical factors such as genetic mutations and underlying health conditions are well-established contributors to infertility, lifestyle choices and environmental factors also play a crucial role [3,4]. They include diet [5], physical activity, stress [6], and exposure to pollutants [7] and can influence fertility either positively or negatively, particularly by affecting ovulation. Ongoing research is focused on understanding the exact relationship between these factors and specific ovulatory disorders [8].

Female infertility can stem from various causes, such as ovarian insufficiency, diminished ovarian reserve (DOR), fallopian tube obstruction, or abnormalities in the reproductive anatomy [9,10]. The importance of ovulatory infertility, accounting for a significant proportion of overall cases, is a critical concept whose importance stems from the fact that ovulatory dysfunctions can often be modulated by modifiable factors [11,12] such as physical activity [13], weight management [14], proper nutrition [15], or alcohol and tobacco use. Addressing these lifestyle factors provides potential avenues for the treatment and management of ovulatory disorders.

The impact of diet and obesity on fertility is also connected with insulin resistance [16,17,18], occurring when insulin in the bloodstream is unable to effectively promote glucose uptake or utilization by insulin-sensitive organs and tissues. Through its connection with both the endocrine system and nutrition—which are in turn involved in processes connected with infertility—and its higher prevalence among women [19], insulin resistance is considered among those factors that need to be taken into account when ovulatory infertility is concerned [20].

Ovulatory disorders, affecting approx. 25% of couples experiencing infertility, are categorized by the WHO into three primary groups [10,21]. Due to the fact that this classification is both rather outdated and used in the literature inconsistently, the authors designed their own classification of the relationships between ovulatory infertility and lifestyle factors [9]. Based on the 1973 WHO classification, this approach offers novel insights into the issue, focusing on the current scientific consensus as to the possible relationships between various factors and conditions present in ovulatory infertility.

The diagnostic approaches for ovulatory infertility typically include obtaining a detailed medical history—focusing on menstrual regularity and ovulation—along with a gynecological examination. Further diagnostic measures often involve ultrasound monitoring of the menstrual cycle and hormonal tests to assess the levels of follicle-stimulating hormone (FSH), luteinizing hormone (LH), estradiol, thyroid-stimulating hormone (TSH), testosterone, progesterone, and prolactin. Most importantly, in cases where diminished ovarian reserve is suspected, anti-Müllerian hormone (AMH) testing is recommended [22,23,24].

The literature data indicate that serum AMH levels provide an estimate of ovarian reserve and are a reliable indicator of the quantity of oocytes [25,26]. However, Cedars [25] emphasizes that AMH must be interpreted in the context of the overall endocrine environment, as conditions such as hypogonadotropic hypogonadism or the use of hormonal contraceptives can lower AMH levels without accurately representing ovarian reserve. Another problem is the absence of an international standard for AMH, which complicates the comparison of different AMH tests [27,28]. Additionally, there is limited understanding of the internal and external factors affecting serum AMH levels, making it difficult to accurately interpret AMH results in clinical practice. Buratini et al. [26] also indicated that differences in the accuracy of fertility predictions are partly due to the modest association between circulating AMH levels and oocyte quality, along with greater variability in basal FSH levels between cycles as women age. Despite these limitations, serum AMH levels remain the marker of choice for assessing ovarian reserve [29] and for this reason it was chosen as the predictor of the presence of ovulatory infertility for the purpose of this study.

It is important to note that age is generally agreed to be one of the most critical factors—or indeed the most critical one—negatively affecting female fertility. Advancing age is closely associated with a decline in reproductive potential [25]. Additionally, poor dietary habits and environmental factors can accelerate the aging process of oocytes, further impacting fertility [30]. Silvestris et al. [4] indicated that both lifestyle-related factors and metabolic disorders, which include obesity and diabetes, negatively impact female fertility by affecting oocyte health and hormonal regulation.

Due to the increasing social and professional pressures, the reproductive age in women has gradually shifted upwards. This delay, combined with the typical components of the modern lifestyle, creates a broader time window for various lifestyle-related disorders and genetic factors to manifest themselves and negatively affect fertility [8,31]. Hence, the importance of modifiable lifestyle factors needs to be emphasized, with a particular focus on dietary intervention that can help preserve ovarian health, improve oocyte maturation, and prevent infertility [4].

Therefore, in order to make it possible to explore the possibilities of influencing female fertility through lifestyle modifications, the authors decided to attempt to establish potential connections between a number of lifestyle-related factors and AMH levels, which reflect the woman’s ovarian reserve. Hence, the aim of this article was to establish the relationships between body composition parameters and the intake of selected nutrients and AMH levels in the context of ovulatory infertility. Moreover, based on the results—although the results of the study must be seen as preliminary due to the relatively small sample size—an attempt was made to propose certain dietary solutions that could have a positive influence on fertility.

## 2. Materials and Methods

This case-controlled study was conducted on 28 women of reproductive age, aged 26 to 42, recruited in Białystok, Poland. The participants were patients from the Reproductive Health Clinic and the Clinic of Endocrinology, Diabetology, and Internal Medicine of Medical University of Białystok. The participants were recruited and underwent the tests planned in the study in the period from September 2023 to May 2024. The data were collected in the same period and analyzed in September 2024.

The inclusion criteria were as follows: age > 18 years and no co-morbid chronic diseases. The exclusion criteria included active pregnancy or breastfeeding, the diagnosis of a thyroid disease, polycystic ovary syndrome, or fallopian tube obstruction, and using hormonal contraceptives. These were established based on patient interviews. Due to the retrospective character of this research, which is based on patient data collected in the clinic during routine operation, no follow-up visits were planned as each patient had their own different health needs, many of them not requiring a follow-up.

Ethical approval for the study was obtained from the Bioethics Committee of the Medical University of Białystok (ethical approval no. APK.002.99.2023, dated 16 February 2023), and the study adhered to all relevant protocols and regulations. Informed consent was obtained from all participants prior to the study’s commencement.

### 2.1. Assessment of Ovarian Reserve Parameters

Levels of the following reproductive hormones were measured: anti-Müllerian hormone (AMH), luteinizing hormone (LH), follicle-stimulating hormone (FSH), prolactin (PRL), estradiol, progesterone, and testosterone. Tubal patency was assessed using sonohysterography (sono-HSG). To rule out thyroid disease, thyroid-stimulating hormone (TSH) levels were also determined. All assessments were conducted by a trained and experienced gynecologist. Blood samples for progesterone measurement were collected during the luteal phase, while samples for other hormonal tests were collected during the follicular phase of the menstrual cycle.

### 2.2. Biochemical Parameters

Fasting glucose, insulin, cholesterol, microelements and other relevant biochemical markers were analyzed. The homeostatic model assessment of insulin resistance index (HOMA-IR)—which is a widely utilized tool for assessing insulin resistance—was calculated to evaluate insulin resistance. It is derived from fasting insulin and glucose concentrations. A HOMA-IR value greater than 1 is typically regarded as abnormal, while a value exceeding 2 suggests the presence of insulin resistance [32]. HOMA-IR was calculated as follows: fasting insulin (μIU/mL) × fasting glucose (mmol/mL)/22.5.

### 2.3. Dietary Intake Assessment

All participants were instructed to complete a 3-day dietary diary. To reflect their typical eating habits, the diary covered two weekdays and one weekend day. In order to ensure data accuracy, a trained researcher was present to verify the reliability of the recorded information. The average nutrient intake was determined based on data from the dietary diaries, and concerned calories, macronutrients, vitamins, and minerals. Data thus obtained were analyzed using Dieta 6 software (National Institute of Public Health, Warsaw, Poland). The results were compared to the dietary reference intake norms for the general Polish population, considering the estimated average requirement (EAR).

### 2.4. Body Composition Analysis

Body composition was assessed using bioelectrical impedance analysis (BIA) using the InBody 720 device. Parameters such as body weight, height, body mass index (BMI), percent body fat (PBF), waist–hip ratio (WHR), and total body water (TBW) were evaluated.

### 2.5. Potential Bias and Confounders

Several variables may act as confounders in this study. Without controlling for all potential confounders, such as lifestyle factors (e.g., physical activity, smoking), socio-economic status, and environmental influences, the results could be skewed. While multivariate regression was used to account for some confounders, it may not capture all the relevant factors influencing ovarian reserve parameters.

The study sample consists of a small group of 28 women, which can lead to sampling bias. Such a small sample size, particularly when drawn from a specific region (Białystok, Poland), may not reflect the broader population of women with reproductive concerns.

Due to the fact that dietary intake assessment was based on the 3-day dietary diary, it is susceptible to recall bias as patients may not accurately remember or may fail to report important dietary habits. The same issue concerns the data used in the study that were sourced from patient surveys and interviews.

As this is a cross-sectional study with no follow-up, its design makes it impossible to establish causality or account for changes over time, i.e., the study is susceptible to hypothetical/temporal bias. The data reflect a snapshot at one point in time, and any temporal changes in participants’ health cannot be assessed.

It is also possible that non-response bias was present in the study, although there are no indicators that those patients who did not decide to participate in this study differed from those that did.

### 2.6. Statistical Analysis

Due to the small sample size and the inability to verify the normality of distribution of the analyzed variables, nonparametric tests were used. Univariate and multivariate linear regression analyses were performed, with serum AMH level as the dependent variable, to investigate the relationships between independent variables and AMH levels. The univariate analysis assessed the individual impact of each independent variable on AMH. The multivariate model is a proposed explanation of changes in AMH levels under the simultaneous influence of selected variables. Only patients whose data were complete were included in the analysis.

Statistical significance was set at *p* < 0.05. The results were processed using Statistica 13.3 (TIBCO Software, Palo Alto, CA, USA) and Stata 18.0 (StataCorp, College Station, TX, USA).

## 3. Results

Comparing lifestyle-related parameters, it was observed that the older age of the participants negatively affected serum AMH levels. The conducted univariate linear regression analysis revealed a statistically significant impact (*p* = 0.02) of age on AMH. In terms of body composition parameters, the conducted univariate linear regression analysis revealed a statistically significant association of all the studied parameters with AMH levels.

Out of the four studied parameters related to carbohydrate metabolism, the conducted univariate linear regression analysis revealed a statistically significant association of only half of those, i.e., fasting glucose (*p* = 0.03) and sucrose intake (*p* = 0.01), with serum AMH levels. The results of univariate linear regression analyses concerning the association between parameters related to lipid metabolism and AMH levels—similarly to the case of parameters related to carbohydrate metabolism—showed statistically significant associations in the case of half of these, i.e., HDL (*p* = 0.03), α-linolenic acid (*p* = 0.02), and *n*-3 acids (*p* = 0.02). In terms of the intake of vitamins and minerals parameters, the conducted univariate linear regression analysis revealed a statistically significant association of all the studied parameters with AMH levels. The results of all the aforementioned univariate analyses are shown in Table 1.

On the basis of the results of the univariate linear regression analyses, the construction of a multivariate model was attempted. Despite the fact that a statistically significant influence on AMH was found in the case of a number of the parameters in the univariate analysis, many of them failed to be included in any of the attempted models. Eventually, a model based on the following five parameters was proposed, i.e., age, WHR, average iodine intake, average sucrose intake, and average consumption of erucic acid (Table 2). The adjusted R^2^ value of the model identifying the independent predictors of AMH serum was 64.18%, which means that over 64% of the variability of AMH is explained by the model.

## 4. Discussion

It must be emphasized that the results of this study confirm that age is negatively associated with serum AMH levels (*p* = 0.02), thus being also associated with ovulatory infertility. This is confirmed in several studies [25,29,33] and aligns with the generally negative impact of age on female fertility [34,35]. Similarly, the negative association between body composition parameters and serum AMH levels is clearly corroborated in the results of this study as all the studied parameters in this group were found to be negatively associated with serum AMH levels. This easily recognizable trend clearly shows that unhealthy body composition can have a detrimental impact on fertility and hence indirectly points to the importance of a healthy diet in the area of women’s fertility. Other studies confirm these relationships, establishing that an elevated visceral adiposity index (VAI) is positively correlated with an increased prevalence of infertility among women in the United States [36], and showing that numerous indicators of VAI are linked to an increased prevalence of infertility [37]. A prospective study performed by Song et al. [38] showed no significant correlation between visceral fat levels and reproductive outcomes; it did, however, find a significant difference in leg body fat mass (LBFM) concerning the distribution of reproductive outcomes. Generally speaking, despite the minor disagreements between studies, it can be safely assumed that body composition parameters, including the visceral obesity index [36], can help identify women facing infertility, and managing visceral obesity may reduce the risk of infertility. Ultimately, though, it remains uncertain at this point how obesity may adversely affect AMH levels and potentially ovarian reserve in otherwise healthy women with regular menstrual cycles [39], thus necessitating the performance of large-scale prospective studies.

In terms of carbohydrate metabolism, the results of the study suggest that the intake of various carbohydrates has a generally negative association with female fertility [40,41,42,43]. Most importantly, the relationship between sucrose intake and AMH levels was found to be statistically significant (*p* = 0.01) as well as the relationship between fasting glucose levels and AMH levels (*p* = 0.03). However, simple carbohydrates have not been found to be significantly associated with serum AMH levels, with *p* = 0.37 and *p* = 0.44, respectively. Another parameter, i.e., the intake of digestible carbohydrates, is also worth mentioning. Its borderline level of statistical significance (*p* = 0.51), considering the small study group, may be treated less conservatively, and thus the result—in line with the aforementioned negative associations—may be interpreted as reflecting the same trend of a generally negative influence of carbohydrates on AMH levels, and thus fertility. On the other hand, the fact that HOMA-IR was not found to be associated with serum AMH levels (*p* = 0.09) may be seen as surprising due to the fact that insulin resistance seems to play an important role in ovulatory infertility [44,45,46,47,48]. One interpretation of this result that seems plausible is that although insulin resistance does play a significant role in fertility, it may not be associated with ovarian reserve and thus AMH levels in particular.

The results of this study confirmed the widely held belief regarding the positive impact of omega-3 acid intake on human health. The consumption of both α-linolenic acid (18:3(*n*-3)) and fatty acids as a sub-group (*p* = 0.02), and *n*-3 acids as a group (*p* = 0.02) were found to be in a positive relationship with AMH levels. A pilot study performed by Lipovac et al. [49] also pointed to the positive effect of omega-3 acids intake on serum AHM levels, although it needs to be emphasized that the study tested a supplement with numerous micronutrients as ingredients, which leads to the high level of confounding. Another study [50], on the other hand, performed in a group of 200 female subjects, found no association between the intake of either omega-3 or omega-6 acids and fertility. Interestingly, these findings are corroborated in this study in regard to omega-3 acids, as no statistically significant association has been found between the intake of omega-6 acids and AMH levels (*p* = 0.84).

The aforementioned uncertainty regarding the relationship between the intake of omega acids and ovulatory infertility is reflected in the results of this study concerning fats in general. It has been found that the intake of saturated fatty acids is not associated with AHM levels, in spite of their overall negative health impacts in other areas. Moreover, the impact of monounsaturated fatty acids, which may have anti-inflammatory effects, is difficult to ascertain. On the one hand, the association between the intake of erucic fatty acid (22:1) with AMH levels has been shown to be slightly above the level of statistical significance (*p* = 0.07) in a univariate linear regression analysis. Due to the relatively small group size that may have influenced the quoted level of statistical significance, i.e., slightly above the cutoff value, and the literature data pointing to its beneficial role in numerous conditions indirectly related to ovulatory fertility [51,52,53] combined with the known beneficial health properties of omega-9 acids in general, it was decided that, if possible, erucic acid should be included in the designed multivariate linear regression model. As it turned out, the proposed model showed that the association of erucic acid intake with AMH levels was statistically significant (*p* = 0.04). Obviously, this cannot be interpreted unanimously as the existence of an impact of erucic acid consumption on fertility, yet it does suggest that such a possibility does exist.

Ma et al. [54] found a positive correlation between vitamin intake and AMH levels. Moreover, they established that women with premature ovarian insufficiency (POI) had significantly lower levels of vitamin E compared to those with regular menstrual cycles. Although the study was performed in a relatively small number of subjects (40 with POI; 56 in the control group) and thus its results must be considered preliminary, they align with the findings of this study, confirming that vitamin E plays a positive role in fertility. The results of another preliminary placebo-controlled study [55], performed in a group of 70 women divided equally into the study and the control group, corroborate this conclusion, finding that taking vitamin E and selenium supplements can prevent the depletion of the ovarian reserve. In addition, the aforementioned pilot study performed by Lipovac et al. [49] also identified vitamin E as one of the micronutrients that may play a role in preventing ovarian reserve depletion. These results are in line with the findings presented in this article, which show a statistically significant (*p* = 0.03) positive relationship between vitamin E intake and AMH levels.

However, it needs to be emphasized that vitamin E is the only compound in the group of vitamins and minerals included in this study that showed a positive association with AMH levels. The others, i.e., thiamine, iodine, and manganese, showed statistically significant (*p* = 0.04, *p* = 0.003, and *p* = 0.02, respectively) negative relationships with AMH levels. The literature data [56] point to the negative role of high iodine intake through its impact on insulin secretion via the role it plays in increasing TSH levels. Through its interaction with thyroid activity, iodine at elevated levels may lead to insufficient insulin production, which in turn can cause insulin resistance and/or diabetes, both of which have an adverse effect on fertility. As far as manganese is concerned, the result of this study contradicts the literature data, as it seems that a diet low in the element in question may in fact increase the risk of anovulation, or the inability of the ovary to release an egg during the menstrual cycle, which can lead to infertility [57]. Similarly, although based on animal studies and thus less definitive, the results of certain experiments [58,59] indicate that the influence of thiamine intake on fertility appears to be positive rather than negative. The conclusions and inconsistencies presented above point to the largely undetermined impact of vitamins and minerals on female fertility and raises the question of whether vitamin and mineral supplementation in pregnant women, if not carefully designed in terms of ingredients, may have an effect opposite to that intended.

Among the variables included in the proposed multivariate model, age was negatively associated with AMH levels, aligning with established research on the decline in reproductive potential with age. WHR also showed a negative association with AMH, suggesting that abdominal obesity may adversely impact ovarian reserve. Sucrose intake was similarly linked to lower AMH levels, supporting evidence that high sugar intake can affect reproductive health. Erucic acid intake, however, showed a positive association with AMH, hinting at the potential role of certain dietary fats in supporting ovarian reserve, while iodine intake suggested a possible negative effect, consistent with research on its influence on thyroid function and fertility.

Cedars [25] pointed out that as far as using AMH levels as predictors for chances of conception is concerned, it must be remembered that the levels of this hormone do not provide any indication of oocyte quality or the likelihood of conception; for this reason, age remains the most significant factor in predicting success rates with fertility treatments. However, although their quality does remain to a certain degree uncertain, it cannot be said to be independent from their quantity. This is because with larger ovarian reserve, there is a greater probability that among those oocytes that eventually mature, a certain number will be characterized by a higher quality, and thus also have a higher potential for conception. Therefore, measuring AMH levels can help estimate the likelihood of pregnancy, while low AMH levels may indicate diminished ovarian reserve and signal that there is limited time left to conceive.

Considering the results obtained in the study and various official dietary recommendations [60,61,62,63,64], the authors decided to propose their own practical dietary modifications that can counteract ovulatory infertility. Obviously, it must be remembered that they are by no means the only possible solutions of this kind. Moreover, given the limitations of the small sample size and case-controlled design, further research—especially larger, prospective studies—is needed to validate these associations and to inform fertility care recommendations. Nevertheless, with these limitations in mind, the authors feel that certain nutritional suggestions can be made.

The findings of the study make it possible to infer that dietary enrichment with fatty marine fish—such as Norwegian salmon, herring, or mackerel—or omega-3 supplementation providing at least 250 mg of EPA (eicosapentaenoic acid) and DHA (docosahexaenoic acid) may support female fertility [65,66]. Additionally, incorporating high-quality sources of monounsaturated fatty acids [5], like olive or rapeseed oil, and foods rich in vitamin E [67], including specific oils, nuts, seeds, vegetables, fruits, and certain spices, could also be beneficial. An emphasis on low-glycemic foods, whole grains, and fiber-rich products, alongside minimizing added sugars, is also recommended [5].

In the context of using products with a low glycemic index, it is recommended to include whole grain, unrefined cereals in the diet and to enrich meals with high-quality protein and healthy fats [68]. This approach can effectively lower the glycemic index of the entire meal, promoting blood glucose stabilization and minimizing the risk of sudden insulin spikes throughout the day [69]. Due to the high glycemic index, refined grain products such as white flour and white bread should be avoided. Additionally, to limit their impact on glycemic levels, it is advisable to eliminate added sugars from the diet.

Current dietary recommendations are usually aimed at the general population, which may limit their effectiveness for specific groups, such as individuals with infertility issues. Nutritional guidelines are often developed to prevent lifestyle diseases, such as obesity [70], or cardiovascular conditions [71]. Considering that infertility affects a significant percentage of the population and constitutes a major health issue [72], creating dedicated dietary recommendations for this group seems to be a reasonable consideration. Moreover, many general dietary guidelines require updating to better address the needs of modern society and take into account specific health challenges.

In light of the above discussion of dietary requirements, viewed in the context of the findings of this study, it needs to be emphasized that supplementation of particular nutrients can be achieved not only through the intake of dietary supplements, but also—or chiefly—through adherence to a well-crafted, balanced diet. Such personalized nutrition plans should be prepared by professionals in accordance with the latest recommendations and scientific knowledge. They can be effective in the management of numerous conditions, and one of the areas in which targeted dietary changes can have a meaningful impact is ovulatory infertility.

## 5. Conclusions

The results of this study provide preliminary evidence that certain modifiable lifestyle factors—alongside non-modifiable factors such as age—can influence ovarian reserve as measured by serum AMH levels and thus have an impact on female fertility. The findings indicated age, higher WHR, and increased sucrose intake as significant predictors of lower AMH levels, while erucic acid intake demonstrated a positive association.

These results suggest that targeted dietary modifications, especially regarding carbohydrate and fat intake, may support ovarian reserve and fertility outcomes. In this light, for a diet to support fertility, it should be enriched with mono- and polyunsaturated fats—primarily omega-3 fatty acids—while reducing overall carbohydrate content, prioritizing low-glycemic-index foods, and minimizing added sugars. Additionally, high fiber content and adequate vitamin E intake are recommended for women planning pregnancy.

Although the results support the research hypothesis that certain lifestyle modifications can impact AMH levels and thus have a positive impact on female fertility, more robust studies are needed to strengthen these results and make targeted well-informed dietary interventions possible. While increased age remains an extremely important, non-modifiable predictor of reduced AMH levels, this study underscores the vital role that modifiable diet-based lifestyle interventions play in fertility management.

### Limitations of the Study

The study’s findings could have been strengthened if the study had been conducted on a larger sample group, given the modest effect size observed for the phenomena under examination. Additionally, the cross-sectional, clinical-control design limits the interpretability of results compared to those from a prospective study, which the authors took into consideration when forming inferences presented in this article. To address these issues, the authors plan to conduct a larger-scale, prospective study as a follow-up. Furthermore, due to the relatively large number of exclusion criteria, many women who initially wished to participate in the study had to be excluded. An additional limitation was the use of the three-day food diary, which is vulnerable to recall bias. The researchers sought to mitigate this by emphasizing to participants the importance of accurate data recall and recording when completing the diary. They also made sure the participants clearly understood all the instructions on how to properly fill in the diary.

## Figures and Tables

**Table 1:** Results of univariate linear regression analysis of the associations between parameters concerning age, body composition, metabolic indicators, and nutritional patterns measured in this study and serum AMH levels.

Variable	Estimate	95% CI		*p*-Value
Age	−0.1635	−0.2919	−0.0352	0.015
***Body composition***				
Weight	−0.0487	−0.0812	−0.0161	0.005
TBW (total body water)	−0.1282	−0.2471	−0.0092	0.036
Protein	−0.4771	−0.9150	−0.0393	0.034
FFM (free fat mass)	−0.0942	−0.1815	−0.0069	0.035
SMM (skeletal muscle mass)	−0.1557	−0.3018	−0.0096	0.038
BMI (body mass index)	−0.1418	−0.2372	−0.0464	0.005
PBF (percent of body fat)	−0.1231	−0.1973	−0.4887	0.002
WHR (waist–hip ratio)	−10.7336	−19.8491	−1.6181	0.023
***Parameters related to carbohydrate metabolism***				
Fasting glucose	−0.0712	−0.1365	−0.0058	0.034
HOMA-IR	−0.2456	−0.5307	0.0394	0.088
Glucose (intake)	−0.0759	−0.2472	0.0956	0.371
Fructose (intake)	−0.0510	−0.1854	0.0833	0.442
Sucrose (intake)	−0.0436	−0.0761	−0.0111	0.011
Digestible carbohydrates (intake)	−0.0088	−0.0176	0.0000	0.051
***Parameters related to lipid metabolism***				
HDL	0.0424	0.0043	0.0806	0.031
Fatty acids (18:0) (stearic acid)	−0.2303	−0.5007	0.0401	0.092
Fatty acids (20:0) (arachidic acid)	7.9235	−1.0740	16.9210	0.082
Fatty acids (22:1) (erucic acid)	2.3790	−0.2335	4.9915	0.073
Fatty acids (18:3) (α-linolenic acid)	0.9317	0.1436	1.7199	0.022
*n*-3 acids	0.7001	0.1380	1.2622	0.017
*n*-6 acids	−0.0253	−0.2775	0.2269	0.838
Saturated fatty acids	−0.0164	−0.0835	0.0508	0.621
***Vitamins and minerals***				
Thiamine	−1.3591	−2.6596	−0.0585	0.041
Vitamin E	0.1563	0.0196	0.2930	0.027
Iodine	−0.0184	−0.0302	−0.0067	0.003
Manganese	−0.4027	−0.7443	−0.0611	0.023

**Table 2:** The multivariate linear regression model that explains changes in serum AMH levels.

Variable	Estimate	95% CI		*p*-Value
Age	−0.1140	−0.2024	−0.0256	0.014
WHR	−10.4748	−16.5392	−4.4103	0.002
Average sucrose intake	−0.0284	−0.0546	−0.0023	0.034
Average erucic acid intake	1.7173	0.0478	3.3868	0.044
Average iodine intake	−0.0097	−0.0198	0.0003	0.058

## Data Availability

The data presented in this study are available on request from the corresponding author.
